# John Chinaman, M.D.

**Published:** 1889-11-09

**Authors:** J. Bowles Daly


### John Chinaman, M.D.

By Dr. J. Bowles Daly.

Medical science in Europe has attained a lofty position, owing to the high character of its professors and students. A glimpse of the same profession in the vast empire of China may prove entertaining, if not profitable, to the readers of The Hospital.

China is a fantastic country, a preposterous combination of solemn nonsense and classical caricature, a sort of spontaneous upside-downness and inside-outness which can neither be exaggerated nor exhausted. In the Flowery Land there are doctors for all kinds of diseases. Specialism is the order of the day.

To begin with, there are doctors for cold diseases and doctors for hot, doctors for diseases of wind, and doctors for diseases of water, for women, babies, and old men. Tchung-tsen, the most eminent medical practitioner in China, is the seventh son of a seventh son, forty-nine doctors in one, forty-nine times muddled. He is a happy compound of pedant, quack, fortune-teller, and spirit-rapper, flavoured with a dash of Confucian priest, "Just for the look of the thing." The Imperial College of Medicine at Peking is not like our College of Surgeons, insisting that her alumni are well grounded in knowledge before sending them into the world. It is little more than an exclusive club for professional mutual admiration, or a convocation of medical referees for arbitrament in case of malpractice. It has neither the power nor the disposition to check quacks; it does, however, punish offenders who are too poor to bribe the functionaries in office. To conclude from this that John Chinaman's education was narrow would be a rash conjecture. The course of reading is so tremendous as to take away one's breath. I shall only specify a few of the leading medical works. These are Chan-shi-thung's "Universal Medicine," Ching-me-thee's "Principal Veins of the Empire of Medicine" (traced by Wang-Keng-theng long before Harvey came into the world, sometime about the birth of Noah's second son), Wang-Shu-Hoo on "The Pulse," Fung-Se-Kan's "Motley Silk Bag of Deep Learning on Diseases," King-Mu's "Natural History of Necessaries," Sing-Po-Ku on "The Nature and Preparation of Medicine," and, finally, Pu-Ken-Mu's on "The Brain and Stomach," continued by his son, Si-Kan-Yung, and illustrated by Kan-Cho-Ko, an astounding compilation, in forty awful volumes, De Omnibus, Anatomical, Obstetrical, Rebus, et Quibusdam, Botanical, Therapeutical Aliis. Having stuffed all the power of this learning into his memory for handy reference, he is prepared to begin practice.

Tchung-tsen does not believe in modesty; he is not content to put his plate on the door for the best of reasons—no door in China would contain all his titles. He has recourse to the daily papers, and advertises as extensively as Holloway, Pears, or the whole fraternity. He does this in a simple, ingenious manner, alluding to himself in the third person. Here is a sample: "Towers are measured by their shadows, and great men by those who envy them. Envy has taken the measure of Tchung-tsen, and found him lofty. The foundation of Tchung-tsen is deep; it is set in the bowels of mystery, and his pinnacle is high; it glows in the light of truth; his feet are planted among the secrets of earth, and his head is lifted among the discoveries of heaven. Envy and deride Tchung-tsen if you are proud and foolish; honour and imitate him if you are humble and wise; for he has wished to promote the good of others, therefore he has secured his own. But do not think to flatter him. Flattery is his wife; he listens to her politely, but does not believe her. He has more roots than branches; he cannot be overthrown by the wind. Only let us invoke that which he has no right to silence—his learning, and publish that which he has no right to conceal—his skill."

Tchung-tsen knows the value of personality, and sets forth his method of securing a practice with frankness. When the immortal worthies first sent forth Tchung-tsen to sprinkle over humanity the waters of healing, he set out hurrahing in his heart and warbling the "Bright blossomed ode." Like a well-bred man, he accepts his commission with modesty and undertakes his duty with confidence. No coolies or asses go before him in a pompous train, panting and groaning under bloated hampers and bursting sacks. His furniture, compact and precious, he carries with simplicity; in his head all the maxims of Whang-tee, the immortal leech, all the prescriptions of Ko-He up his sleeve, all the charms of Fum-Ko in his queue, all the golden simples of Few-Kun in his blue bag, and the pearl pills and ruby plasters of Hu-Kek-Ne in his pocket.

Tchung-tsen neither advertises nor juggles; his talents are their own sign. When you seek him you can find no other doctor, though a thousand get in the way. Where there is musk, there will be perfume; to smell it, one need not stand in the wind. Tchung-tsen is no blind fowl, pecking at random for worms; his knowledge is sure. He does not climb a tree to hunt for a fish, nor turn over the liver to hunt for diseases of the lungs. He does not send you an olive on the pate of a Buddhist priest, nor engage to perform impossible cures, or turn somersaults in an oystershell. He is no toad in a well, contemplating a patch of sky; the strong, calm eye of his philosophy surveys the universe as from a dome, and takes in at a glance all the demonstrations of science, the delusions of ignorance, and the devices of imposture. He knows that all errors have their brief season; that after a hundred million of lies, the smallest truth remains precisely what it was before; and so he waits, and smiles. And his charges are very moderate.

Diseases, when he calls them, answer to their names, and spirits, vapours, elements, and forces assert themselves before him, like feathers under the fingers of the flower-maker. At his bidding, disorders the most complicated resolve themselves into their several members, and have each a tongue to tell him what they mean. As his large benevolence knows no distinction of persons in the ranks of the afflicted, so his conscientious genius appoints no degree of interest to the various styles of disease, but applies itself with equal science and concern to the bunion on the big toe of the mouse-catcher and the cataract in the eye of a mandarin. The memory of Tchung-tsen is infallible, and the dimensions of his nose are conformable with dignity; his heart is tender, and his fist is spherical; his speech is impressive, and his spectacle-glasses, set in tortoise-shell frames, are two inches and a half in diameter; the length of his queue is regulated by the exactions of public opinion. He has an auspicious mole under his left eye, and his charges are very moderate. On the subject of anatomy Tchung-tsen has some startling views, even from a Chinese point of view. For example, he holds that there is a difference between arteries and veins; that in most Chinese subjects the blood is conveyed by these in opposite directions, not always downward by the arteries nor always upward by the veins; that the heart is a part o the machinery by which this hydraulic process is carried on, and that, under certain circumstances, depending upon the disposition of the five rulers, the blood undergoes a change in passing through the lungs. He has one name for the brain, and another for the spinal cord. He has, also, a pulse for every organ but the brain.

His great theory is that every organ of the body is allied to one of the five elements—earth, wood, metal, fire, and water, which are either hot, cold, moist, dry, or windy. These, again, correspond to the five directions—middle, east, west, south, and north, and are represented by the five colours—yellow, green, white, red, and black.

Thus, the heart, being allied to the element fire, corresponds to the direction south, and is represented by the colour red. Consequently, all derangements of the heart must proceed from excess of the principle of heat and dryness, and should be treated with black medicines, corresponding to the direction of the north, and representing the element of water. And the bowels, being allied to the element earth, corresponds to the direction middle, and are represented by the colour yellow. Consequently, all disorders of the bowels must proceed from excess of the principle of wind, and should be treated with medicines compounded of black, red, green, and white ingredients, corresponding to the direction—north, south, east, and west, and representing the elements—water, fire, wood, and metal. A lovely system! So natural in its simplicity and harmony that in theory it reads like an idyl, and in practice it must be one of the pleasures of imagination to be killed by it.

Tchung-tsen's theory of the pulse is equally novel. He holds that there are different pulses, corresponding to the heart, lungs, liver, and all the other organs, and that, to feel the pulse scientifically, you must feel them all, one after the other, and sometimes several together, in order to determine their several relations. Tchung-tsen plays on his patient's twenty-four pulses with all his fingers, and maintains a protracted telegraphic correspondence with his twenty-four insides. The materia medica of Tchung-tsen is delicious. Asses' glue and birdnests are mild tonics, stag's glue, dog's flesh, and walnuts strengthen the kidneys, iron filings, loadstone, and silverleaf repress weakness, camphor, centipedes, snake's skin, and tiger's bones disperse wind, soapstone, amber, and red hairs are laxatives, water-melon, bamboo-shavings, verdigris, and warm water are cooling purgatives.

In China the medical profession is neither glorious nor lucrative. The visits of the doctor are not charged for at all. His complicated "simples" are sold cheap, and always on credit. It is also a custom of the country not to pay for medicine which the patient may fancy has done him no good. Poor Tchung-tsen often spends three pounds to collect one, and if the patient is so thoughtless or inconsiderate as to die, his sudden departure may be the death of his medical adviser.

